# Stable Liposome in Cosmetic Platforms for Transdermal Folic acid delivery for fortification and treatment of micronutrient deficiencies

**DOI:** 10.1038/s41598-018-34205-0

**Published:** 2018-10-31

**Authors:** Mudra Saurabh Kapoor, Anisha D’Souza, Noorjahan Aibani, Swathi Sivasankaran Nair, Puja Sandbhor, Durga kumari, Rinti Banerjee

**Affiliations:** Department of Biosciences & Bioengineering, Indian Institute of Technology Bombay, Powai, Mumbai, 400076 India

## Abstract

Oral folate fortification has been successful in many developed nations, however, developing countries still face low compliance and high incidence of folate deficiency associated with low birth weight infants and preterm deliveries. We report safe and efficient approach for transdermal systemic folate delivery using fluidising liposomes (120 ± 4 nm) stabilised within 3D matrix of naturally occurring cosmetic bases: Fuller’s earth and henna with room temperature stability. The proof of stratum corneum fluidisation was established *ex-vivo* by Langmuir-Blodgett film, FTIR and confocal imaging in rat skin. *In-vivo* topical application in rats showed 11-fold increase in plasma folate within 2 hr, confirming systemic delivery through skin. Efficacy study in folate deficient rats over 4 weeks showed significantly higher plasma levels compared to oral delivery with significant skin depot. Sub-acute toxicity studies in rats at 750-fold higher doses showed safety after 4 weeks daily application. Primary irritation patch test on 25 healthy human volunteers proved non-irritant nature of the nutricosmetics. The technology is first demonstration of transdermal folate fortification with nanosized liposome incorporated in cosmetics, without synthetic surfactants/ethanol or need of external energy. The platform technology opens the possibility of delivering multiple nutrients systemically through skin and can be scaled for affordable community fortification.

## Introduction

Folates are essential for maintaining healthy growth of erythrocytes, DNA repair and various metabolic reactions in the body. The Recommended Dietary Allowance of folic acid is 0.4 mg per day during pregnancy and its requirement rises during periconceptional period^1^. Poor micro nutritional status of folic acid leads to “hidden hunger”, where the deficiency is not overtly manifested but is linked with significant reproductive risks such as infertility, fetal structural defects leading to premature babies and low birth weight; long-term diseases like macrocytosis, megaloblastic anemia and neural tube defects^2^. Folate fortification programs are run in various countries to counteract this issue and have been successful in developed nations^3^. Insufficient dietary intake of folic acid has been a major concern amongst the general population specifically in African^4^ and Asian^5^ subcontinents. There are benefits to the fortification of folate amongst young girls and women of childbearing age, not just at the time of pregnancy, which may often be unplanned^6^.

Rural or uneducated masses are often hesitant to take oral medication for nutritional deficiency due to their cultural and socioeconomic differences and lack of health awareness^7^. Differences in food preferences and interactions, low stability of folic acid on storage at room temperature^8^, extensive liver metabolism, and pregnancy-induced vomiting often limit oral fortification.

The present study describes for the first time, nanosized liposomal folate consisting of soya phosphatidylcholine and oleic acid loaded cosmetics that are cholesterol and ethanol free and have enhanced stability at room temperature without the need for lyophilisation. Transdermal strategies using liposomes have been limited due to low stability and low transdermal permeability of liposomes, and often require ethanol, synthetic surfactants like Tween that can be irritant to sensitive skin types. Other approaches include using iontophoresis or sonophoresis which require external sources of energy for permeation through skin. These disadvantages are overcome in the present study by developing encapsulated nanoliposomes of phosphatidylcholine and fatty acids stabilised within a cosmetic base for transdermal delivery of folate. The technology does not require synthetic surfactants nor does it require the presence of external sources of energy like iontophoresis for enhanced transdermal penetration. The three-dimensional matrix of the cosmetic base causes enhanced stability at room temperature. Transdermal delivery of folic acid using nanosized liposomes in cosmetics has not been explored so far.

Keeping cultural and economic issues in mind, we have evaluated various forms of cosmetics such as Fuller’s earth, Henna and lotion that are easily available and are socially acceptable amongst Asian and African women and are also inexpensive. Lipid vesicles are formulated with varying concentrations of GRAS approved, soya phosphatidylcholine (SPC) and fluidizing fatty acids for delivery of folic acid. Proof of concept was achieved by permeation study of folate liposomes across excised rat skin and further *in vivo* efficacy studies in folate- deficient rat models. A depot is formed within the skin which releases folic acid in a controlled manner even after wiping the delivery system from the skin surface in 10 minutes. The fluidizer combination alters the stratum corneum (confirmed by *in-vitro* FTIR data) without affecting the long-term barrier function of skin (confirmed by *in-vivo* acute toxicity study). The safety of the folate delivery carriers was established *in-vivo* at 750-fold high doses of 5 mg/kg folate. Dermatology safety evaluation conducted on 25 healthy human volunteers proved skin compatibility on human skin. The technology can act as a platform for the transdermal delivery of various micronutrients through the skin using fluidizing liposomes incorporated within standard cosmetic bases.

## Results

### Development and optimisation of Nanosized stable Liposomes for dermal penetration

**Figure 1:**
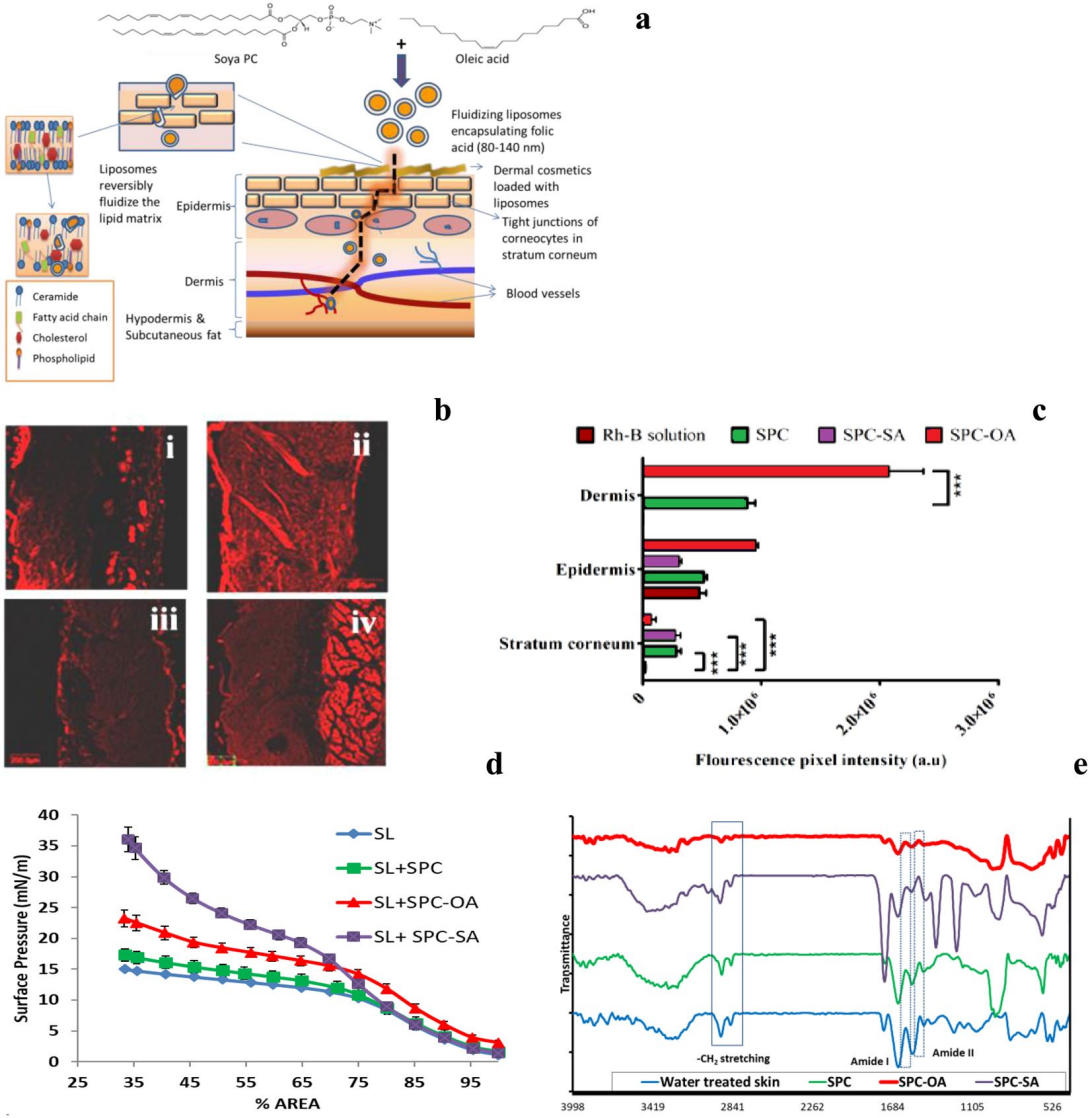
Liposomes for Transdermal delivery. (**a**) Schematic representation of Fluidization of stratum corneum by folate loaded liposomes incorporated in cosmetic base. (**b**) Confocal laser microscopy of rat skin treated with Rhodamine-B:i- Rh-B solution(control), ii-SPC, iii-SPC-SA, iv SPC-OA. SPC, SPC-SA, SPC-OA liposomes encapsulating Rhodamine-B, Scale bar = 200  µm, SPC shows follicular penetration, SPC-SA does not show any penetration in dermis, SPC-OA shows highest penetration beyond dermis. (**c**) Fluorescence intensity graph showing depth of penetration of Rh-B loaded liposomes ***P < 0.001 by student t-test. SPC-SA shows no dermis penetration, SPC-OA shows maximum penetration into the dermis. (**d**) Surface pressure- area isotherm of SL, SPC, SPC-OA and SPC-SA mixture at 37 °C. (**e**) ATR-FTIR spectra of blank rat skin (untreated) and post treatment with SPC, SPC-SA and SPC-OA liposomes representing molecular shifts in lipid chains of stratum corneum, kinked structure of oleic acid can efficiently disrupt the ordered packing temporarily and diffuse across the lipid bilayers of stratum corneum. (**b**–**d**) n = 3. Mean ± SD.

Our study aims at delivering folic acid into systemic circulation via the skin. We, therefore, prepared nanovesicles to reversibly fluidize the outermost stratum corneum, transverse through different layers of skin and reach the blood vessels present in dermis carrying its cargo safely (Fig. 1a). Soya phosphatidylcholine, a natural unsaturated phospholipid was chosen to develop a flexible liposome for fluidising skin. In addition, 18 carbon fatty acids were used in saturated form as stearic acid- (SA) and in unsaturated form as Oleic acid- (OA). The depth and extent of penetration through different layers of skin was compared using Rh-B labeled liposomes for 24 hr by Confocal Laser Scanning Microscopy (CLSM) images **(**Fig. 1b**)**. SPC-OA enhanced penetration of Rh-B through intact skin throughout the viable region in channel-like regions and deposited in the deeper dermis layer. It is notable that SPC (size 170 ± 8.9 nm) with similar size as that of SPC-OA (120 ± 4.1 nm) resulted in lower amounts of fluorescence i.e. 0.88*10^6^ a.u. in dermis and 0.51*10^6^ a.u. in the epidermis as compared to 2.07*10^6^ a.u. and 0.95*10^6^ a.u. of SPC-OA respectively (*P* < 0.001) **(**Fig. 1c**)**. In addition, SPC-SA with a vesicle size of 280 ± 14.1 nm resulted in fluorescence up to epidermis only (0.28*10^6^ a.u. in stratum corneum and 0.31*10^6^ a.u. in the epidermis). Fluorescence intensity of SPC-OA was 2.35–fold higher than SPC in the dermis and significantly higher compared to SPC-SA. On the other hand, deposition of SPC-SA and SPC liposomes in the stratum corneum was high signifying its advantage in topical applications. Rh-B labeled SPC-SA ≥ 200 nm could not penetrate well beyond the epidermal layer. Thus, vesicle size and chemical composition of liposome significantly influenced the accumulation of Rh-B in different skin layers. Effect of penetration due to the liposomes was in the order SPC-OA > SPC > SPC-SA.

### Fluidization of stratum corneum

The Langmuir monolayer technique was used to analyse the effect of fluidizing fatty acids on the packaging of stratum corneum lipids (SCL). Langmuir monolayers provide a precise method of evaluating membrane packing with low sample requirement, and allows simulation of biological conditions^9^. We assessed the surface activity profile of SCL alone and in combination with SPC, SPC-OA (9:1) and SPC-SA (9:1). Figure 1d depicts the surface pressure area isotherm of SCL with different chemical mixtures. It can be noted from the figure that the tensiometric profile of SCL and its mixture with SPC, SPC-OA, SPC-SA is different depending upon the composition and saturation of lipids. On film compression all the monolayers show a sigmoidal increase in surface pressure indicating molecular ordering on film compression. All the values here are relative areas as the composition of skin tissues are complex which hinders the direct comparison with the area occupied by individual molecules. A significant shift in the isotherm was observed with the SPC-OA as compared to SPC alone, indicating the higher effect of OA on surface activity profile of SL. A higher surface pressure was obtained for SCL monolayers in the presence of stearic acid at the same area of compression as that of the SCL monolayer in the presence of OA. This suggests rigid molecular packing of stearic acid due to hydrocarbon saturation as compared to oleic acid. Extrapolation of the final steep linear region of isotherm at end compression versus % area axis gives relative limiting area value^10^. From (Fig. 1d) the relative limiting area calculated were 75%, 80% and 92% for SCL, SPC, and SPC-OA respectively. The relative limiting area indicates molecular packing of the film as it is compressed at a constant rate. A higher relative limiting area value denotes lower condensation potential of the film on compression. The data indicate the highest packing in the presence of SCL and modulation of the packing in the presence of SPC, SPC-OA and SPC-SA. The most fluid state (less closely packed) monolayers were seen in the case of SCL in the presence of SPC-OA. This indicates the potential of SPC-OA to act as a fluidiser of the stratum corneum and as a penetration enhancer.

### Modulation of stratum corneum packing

The fluidising properties of SPC, SPC-SA, and SPC-OA liposomes on transdermal delivery through the excised rat skin were studied by ATR-FTIR (Fig. 1e). Ceramide structure of rat skin shows structural resemblance to human skin and it can act as a prototype for *in-vitro* transdermal studies^11^. The stretching bands of -CH_2_ symmetric (2869 cm^−1^) and asymmetric (2935 cm^−1^) confirmed the trans-conformation of lipid hydrocarbon^12^ and C=O of fatty acids (1756 cm^−1^) of untreated rat skin. Change in either peak intensity/area or shift of -CH_2_ stretching is observed when a penetration enhancer interacts with lipid from the stratum corneum^13^. SPC induced a decrease in peak intensities at C=O stretching pattern from the fatty acids near 1745 cm^−1^. SPC-SA vesicles on SC disturbs the C=O stretching at 1751 cm^−1^ but no significant change in lateral packing of alkyl chain of lipids. The saturated SA has limited flexibility and that hinders its penetration potential in the SC. No change in amide zone was observed. This suggests its inability to cause architectural changes in keratin molecules and packing of lipid chains.

Hydroxy stretch bands of water at 3500-3200 cm^−1^were found to be more intense and sharper for SPC-OA vesicles treated skin than that of untreated skin. This suggests increased hydration and fluidity with increased rotational freedom of lipid acyl chains^14^. The amide I (1654 cm^−1^) and amide II (1556 cm^−1^) groups at the head of keratin/ceramides forms a hydrogen network imparting tightly bonded impermeable barrier of the untreated skin. The intermolecular interactions between the amide groups present in the different layers cause a split in the peaks^15^. SC packing modulation is proved by shift in the amide I vibration (anti-parallel β sheets/C=O stretching) from 1654 cm^−1^ to 1672 cm^−1^ in SPC-OA liposomes treated skin. This can be credited to change in the microenvironment of skin with high fluidization^16,17^. Also, amide II vibration (α helix sheets/C-N stretching) at 1579 cm^−1^ with significant decrease in peak intensity was observed. Significant modulation of stratum corneum packing by SPC-OA was confirmed by ATR-FTIR. This formulation was used in further studies for transdermal delivery of folic acid.

### Design of folic acid liposomes (FAL) for dermal penetration

**Figure 2:**
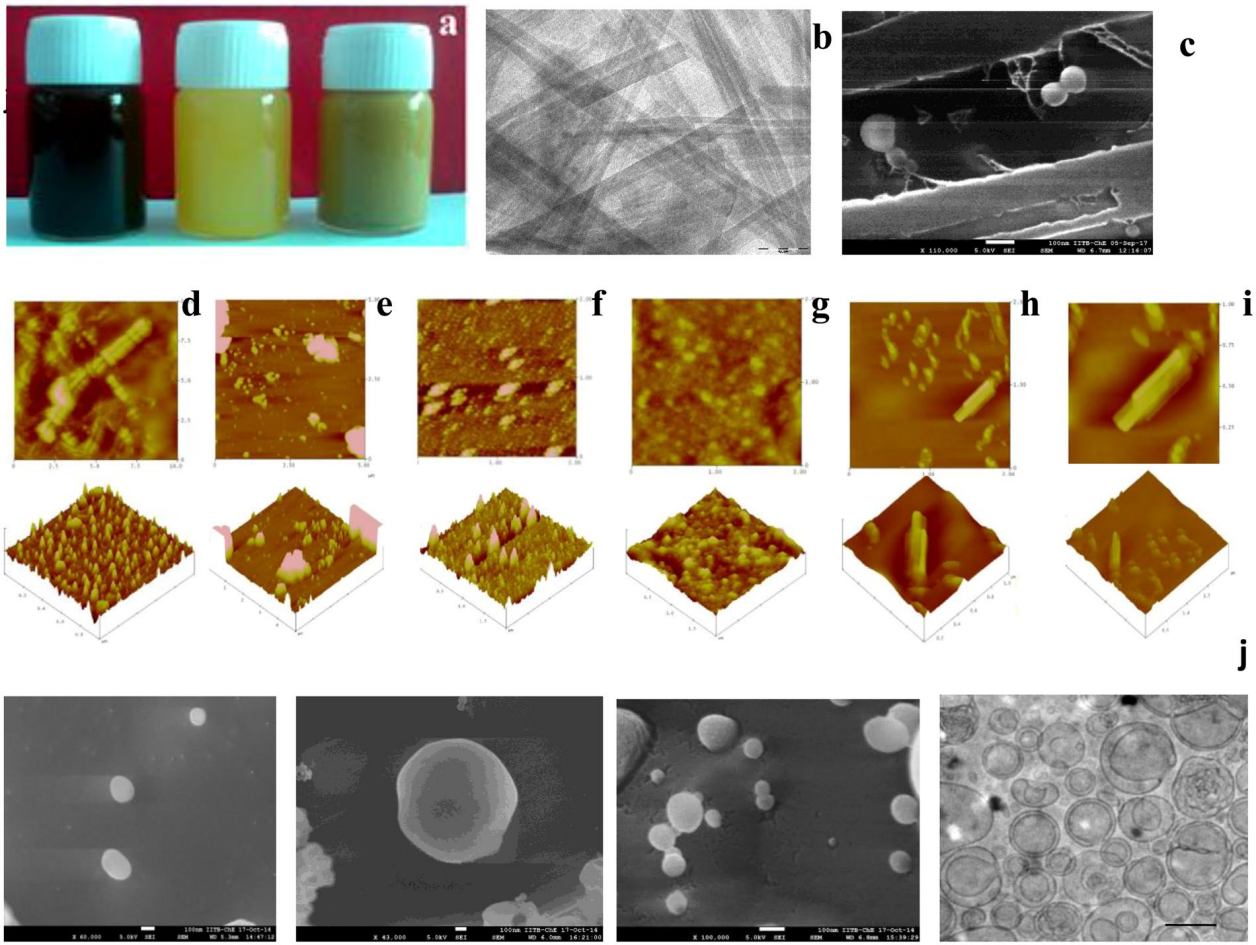
Liposomes in Nutricosmetics. **(a**) [L-R] FAL-H, FAL-R and FAL-MM (50 mg Folic acid/gm cosmetic). (**b**) TEM of MM shows nanorods. (**c**) FEG-SEM of FAL-MM shows distribution of spherical FAL amongst nanorods of MM. (**d,f,h**) Day 1-AFM of FAL-R, FAL-H and FAL-MM. (**e,g,i**) After 6 months-AFM images of FAL-R, FAL-H and FAL-MM. Liposomes carried in 3D matrix of cosmetics prevents its aggregation and enhances stability of liposomes at room temperature. (**j**) Spherical Surface morphology and size of Liposomes (L-R) cryo FEG SEM of SPC (100–150 nm), SPC-SA (>500 nm), SPC-OA (80–120 nm), cryo HR-TEM of FAL respectively. Unilamellar small liposomes of folic acid were obtained with SPC-OA. Scale bar = 100 nm.

Maintaining a final lipid composition of 4 mg/ml and 2:1 w/w (lipid: FA) led to the formation of FAL with 10 ± 1.6% FA encapsulation efficiency, 120 ± 4.1 nm size and negative stable surface charge of −27 ± 3.1 mV. This data was reproducible and validated in intra-day and inter-day variation; liposomes alone were stable at 4 °C for 6 months. 4:1 w/w (lipid: FA) showed 70 ± 3.3% FA encapsulation efficiency, 198 ± 0.2 nm size with −14 ± 2.5 mV surface charge leading to poor stability. On storage at 4 °C, it formed agglomerates and was rejected for further study. 6:1 w/w (lipid: FA) showed 40 ± 0.48% FA encapsulation efficiency, 260 ± 0.4 nm size with −46 ± 6.5 mV surface charge. 8:1 w/w (lipid: FA) showed 40 ± 0.48% FA encapsulation efficiency, 260 ± 0.4 nm size with −46 ± 6.5 mV surface charge. Cutaneous absorption is influenced by vesicle size wherein small vesicles improve skin hydration and result in better penetration. Therefore, lipid composition of 4 mg/ml and 2:1 w/w (lipid: FA) was chosen for further experiments. However, simultaneously encapsulation efficiency is compromised with smaller particle size (Supplementary Table 1). The entrapment achieved was sufficient for developing folate liposomes into cosmetics as per RDA requirement. Developed FAL was characterized by different imaging techniques, illustrating spherical, and unilamellar lipid nanovesicles (Fig. 2).

### Incorporation of FAL into cosmetics and stability of Liposomes in Cosmetic

While the folic acid loaded liposomes alone were stable at 4 °C, attempts were made to further stabilise them at room temperature by incorporation within various cosmetic matrices. Different cosmetics such as body lotion (rose oil-FAL-R), skin coloring agent (henna-FAL-H) and face pack (Fuller’s earth-FAL-MM) were studied as the matrices for incorporation of FAL for transdermal delivery. The composition of the nutricosmetic is described in the methods. Elemental composition of Fuller’s earth consists of Silicate salts of Ti, Fe and Al (Supplementary Fig. 1). Macroscopic image FAL cosmetics comprising micronutrient-loaded FAL are depicted in (Fig. 2a). Rheology studies were conducted to determine the optimum nutrient to cosmetic ratio considering the viscoelasticity of the cosmetics alone (marketed formulation) as standards (Supplementary Table 2). All cosmetics exhibited non-Newtonian flow and shear-thinning characteristics. The viscosity profiles were like that of the cosmetic base alone even after incorporation of liposomes. Thus, suggests the ease of spreading and application of the cosmetic. TEM image revealed the distribution of FAL with size approximately 100–200 nm dispersed in the cosmetic bases (Fig. 2j). AFM of cosmetic-loaded FAL depicted smooth and spherical vesicles of 100–200 nm size indicating uniform distribution of FAL in the cosmetic bases (Fig. 2d–i). The results suggest the feasibility of uniformly loading folic acid loaded liposomes within the cosmetic bases without any change in the rheological parameters. The folic acid loaded liposome incorporated cosmetics were stored at room temperature to determine their stability. After a period of six months, the samples were evaluated for colour, viscosity, presence of liposomes within the cosmetic base and the content of folic acid. TEM and AFM studies (Fig. 2) of the samples after storage clearly indicate intact vesicles distributed throughout the cosmetic matrix. Less than 5% of folic acid leached from FAL-MM after 6 months. All liposomes were 100–200 nm in size and the content of folic acid remained unaltered. The results suggest that the liposomes in cosmetic base were stable at room temperature over a period of six months.

### Proof-of-concept for dermal permeation of nutrient via cosmetic

**Figure 3:**
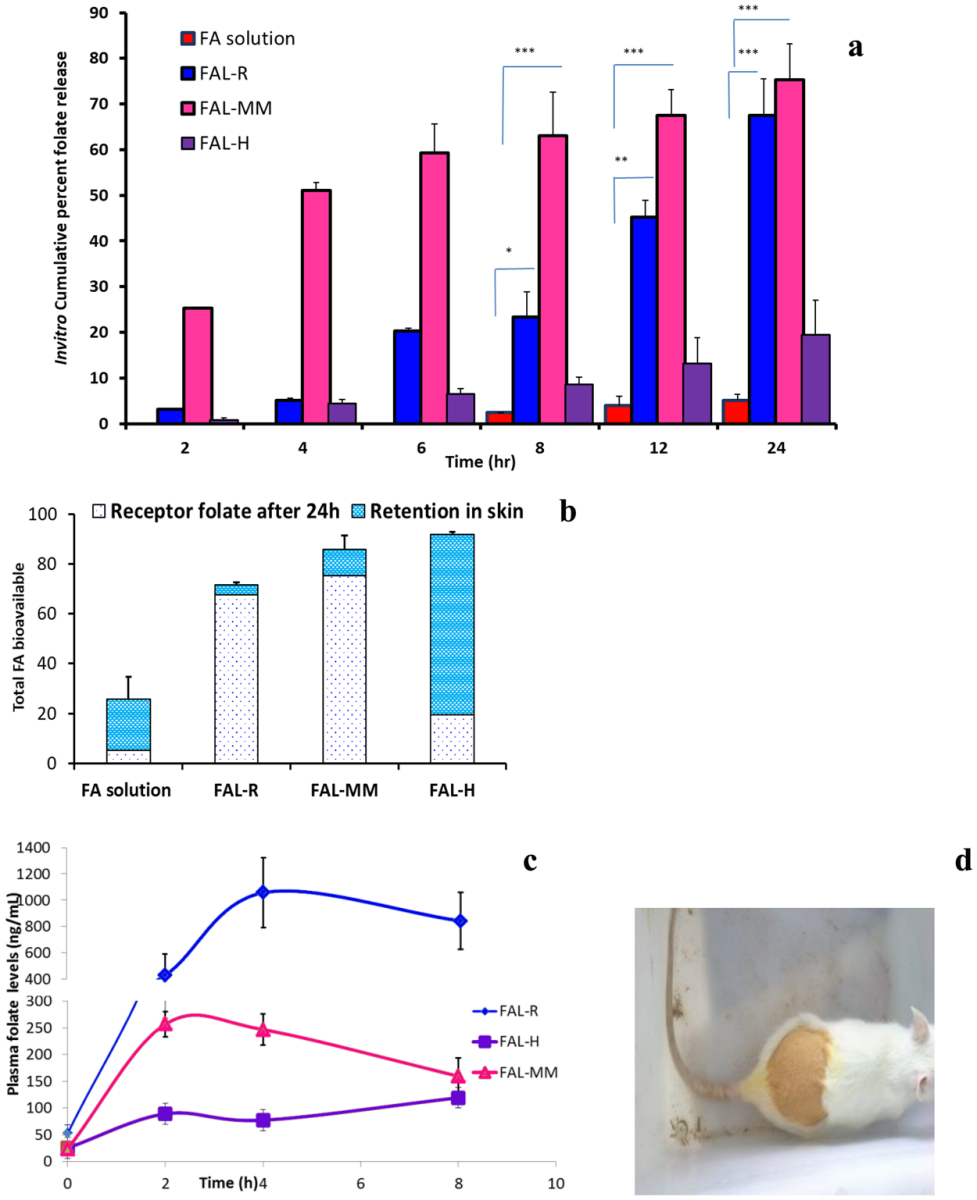
*In vitro* and *In vivo* Folic acid (FA) release performance from Nutricosmetics. (**a**) *In vitro* release of FA (100 µg dose/20 mm^2^) from excise rat skin in 24 hr, ***P < 0.001, **P < 0.05 and *P < 0.01, Student’s t-test. (**b**) Cumulative release of folic acid from different formulations and subsequent skin depot obtained after removing the formulation after 24 hr shows one compartment first order kinetic release. FAL-R shows 70% release of FA, FAL-MM shows 80% release of FA, FAL-H shows 20% FA release but has greater ability to form skin depot, Folic acid solution being hydrophilic has poor permeability across SC. (**c**) Biopharmacokinetic release of single FA (1 mg dose per rat) in healthy Sprague Dawley rats shows first order release kinetics. FAL-R shows highest release of FA, followed by FAL-MM and FAL-H. The normal folate levels in rats are between 5–30 ng/mL, various formulations led to multifold increase in plasma folate levels. Excessive water soluble folic acid is naturally washed out of the body. (**d**) Representative image of rat after application of Nutricosmetic. N = 6. Mean ± SD.

*In vitro* skin penetration of FAL-loaded cosmetics using Franz diffusion chamber, showed significant amounts of folic acid permeating through skin and were detected in the receptor fluid after a lag time of 2 hr. FA solution showed lag time of 6 h (Fig. 3a**)**. FAL penetration was 8 folds higher compared to FA solution (*P* < 0.05). The steady-state transdermal flux for FA incorporated as FAL-R, FAL-H, and FAL-MM were 17.63 ± 3.26 µg/hr/cm^2^, 27.32 ± 5.38 µg/hr/cm^2^ and 28.34 ± 3.01 µg/hr/cm^2^ respectively. FA solution provided significantly lower flux values of 1.46 ± 0.402 µg/hr/cm^2^ with a lag time of 6 hr (*P* < 0.001). Permeability coefficient of FA solution, FAL-R, FAL-H, and FAL-MM were 0.015 ± 0.003 cm/hr, 0.18 ± 0.02 cm/hr, 0.27 ± 0.016 cm/hr and 0.28 ± 0.05 cm/hr. Occlusive application of liposomes onto skin surface prevents partial dehydration by evaporation. As a result, liposomes tend to penetrate by hydration gradient through deeper sections of skin which are more hydrated. Overall, the total FA available from each system calculated from cumulative % release was FAL-MM > FAL-R > FA-H (Fig. 3a). Overall skin depot was high in FAL-H which can act as a reservoir after removal of cosmetic (Fig. 3b). No penetration of fuller’s earth (monitored as silicate levels) was observed over the 24 hr of permeation studies. The results suggest an increased penetration of folic acid when folic acid loaded cosmetics were applied transdermally.

### Evaluation of transdermal folate fortification *in vivo*

Pharmacokinetic profile of transdermal folate delivery indicated that a single topical application of FAL-R on the skin of female Sprague Dawley raised plasma folate to about 20 folds higher (1057 ng/mL) at 4 hr than that of basal folate levels (52.38 ± 7.37 ng/mL). Single application of FAL-MM showed 11 folds increase (256 ± 29.3 ng/mL) at 2 hr compared with fasting basal level (23.4 ± 1.41 ng/mL) at 0 hr. FAL-H showed 4.7 folds increase (119.26 ± 89.49 ng/mL) at 8 hr compared to fasting levels (25.01 ± 5.35 ng/mL). FAL-R, FAL-H and FAL-MM indicated area under the curve (AUC_0–8_) of 5766 ± 1445, 751 ± 480 and 1597 ± 190 ng/mL.hr respectively, which were significantly higher than basal folate AUC_0–8_ levels of 254 ± 65 ng/mL.hr in normal rats (*P* < 0.001) and a T_max_ of 4 hr, 8 hr, and 2 hr respectively (Fig. 3c). Clearance (CL) of FAL for cosmetics (FAL-H and FAL-MM) were significantly higher than FAL-R with CL of 0.18 ± 0.05 mL/h (*P* < 0.001) whereas volume of distribution for FAL-R, FAL-H, and FAL-MM was 3.11 ± 0.7 mL, 10 ± 4.7 mL and 3.4 ± 0.2 mL with no significant difference between the different groups. No significant difference in AUC, clearance, and volume of distribution among the cosmetic groups (FAL-H and FAL-MM) indicate that cosmetics acted as an inert carrier.

These outcomes validate that there is significantly enhance in plasma folate levels with transdermal folic acid loaded liposomes incorporated in cosmetics.

### Efficacy evaluation of Nutricosmetics in anemic animals

**Figure 4:**
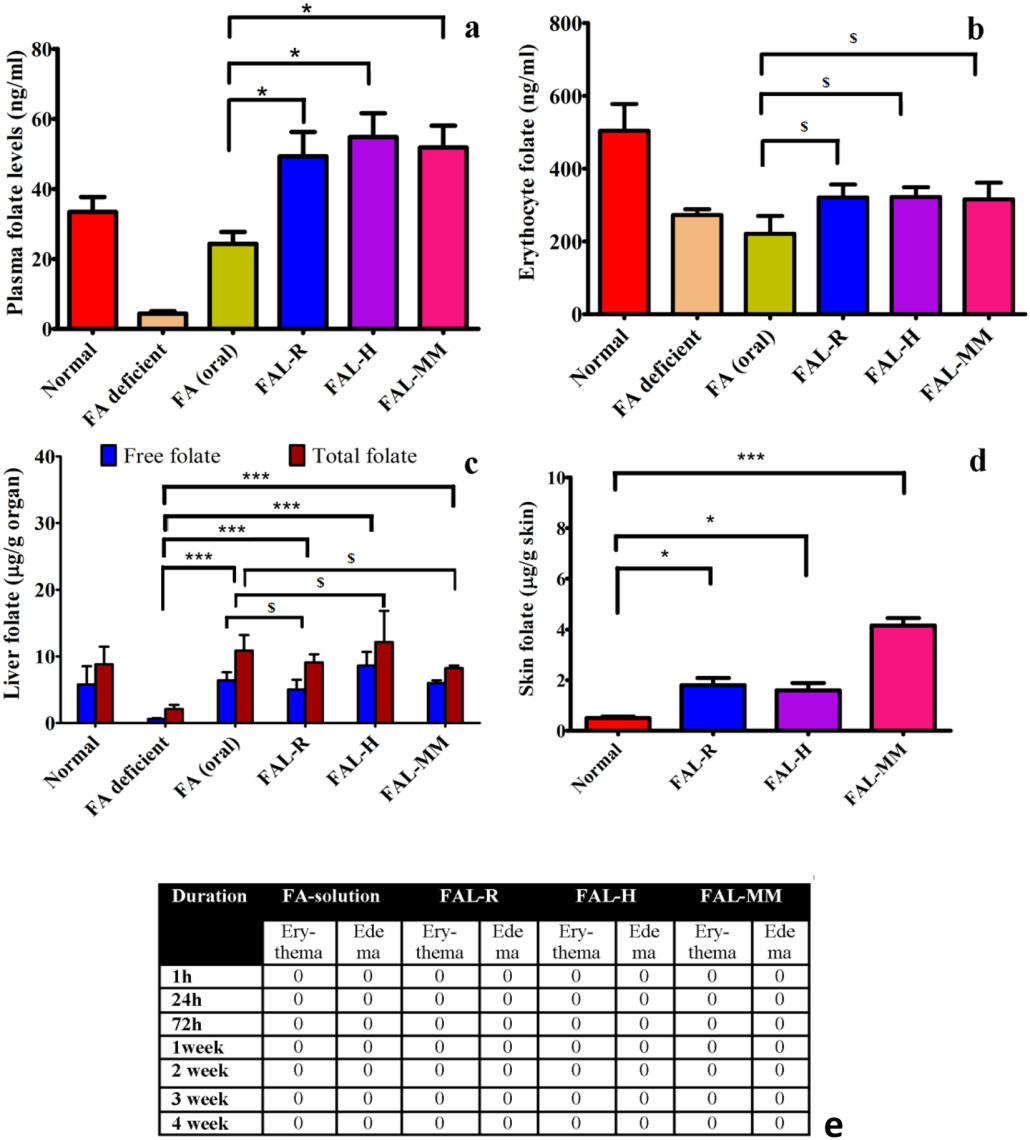
Efficacy study in folate deficient anemic rats. Depletion model was created in rats by feeding them on folate deficient diet. The plasma folate levels dropped to 4 ng/mL in 6 weeks after which daily repletion began for 4 weeks with FA 1 mg/kg dose. Cosmetics were applied to shaved skin and removed after 5–10 min. Normal rats were given regular diet. All other groups were given folate deficient diet. FA deficient group is control group. (**a**) Plasma folate levels after 4 weeks repletion in anemic rats shows significant improvement in folate levels. (**b**) Folate concentration in erythrocyte showed significant folate levels between oral and transdermal delivery. (**c**) Folate concentration in liver showed statistically significant increase in oral and transdermal delivery compared to anemic model. (**d**) Folate depot in skin was highest with FAL-MM, significant depot with FAL-R and FAL-H compared to normal group. ***P < 0.001, ^$^P<0.05 and *P < 0.01, Student’s t-test. (**e**) Numerical scores as per Draize scale of Nutricosmetics after application through the efficacy studies showed no skin irritation or inflammation after regular application. (**a–e**) N = 6. Mean ± SD.

Folate deficiency model was established over 6 weeks with folate-deficient diet. The plasma folate levels dropped to 4.3 ± 1.2 ng/mL and were considered anemic (Supplementary Fig. 2). Efficacy of FAL-loaded Nutricosmetics was evaluated after daily application of FAL-R, FAL-H, FAL-MM to treat folate-deficient anemia in rats for 4 weeks. Folate dose of 1 mg/kg applied transdermally restored the plasma levels above normal after repletion (Fig. 4a) and were significantly higher than that of oral folic acid (*P* < 0.01). Basal folate levels in normal rats (fed with normal diet) were 33.4 ± 7.4 ng/mL. Following repletion with FAL loaded cosmetics, erythrocyte folate levels attained the normal basal folate values with no significant difference amongst FAL-H, FAL-R and FAL-MM applied at the same equivalent dose (*P* = 0.37), oral and transdermal showed equivalent erythrocyte folate levels (Fig. 4b). Liver folate acts as the major reservoir for folate. The free and total liver folate decreased in folate-depleted rats (*P* < 0.05), which significantly improved during repletion period on the application of FAL-loaded cosmetic. The oral and transdermal showed equivalent liver folate repletion (Fig. 4c). On the application of FAL-loaded cosmetics, significant depot of FA was also formed in the skin (Fig. 4d). Neither cosmetic- loaded FAL nor FAL revealed any visually noticeable skin irritation, edema, discomfort or erythema on or around the area of application (Fig. 4e). The results suggest the feasibility of replenishing folate levels in plasma on daily transdermal application of the folic acid loaded cosmetics for a period of 28 days. The nutrient-loaded cosmetics were also non-irritant and safe for topical application, with no adverse histological changes in the tissues.

### Sub-acute repeat dose toxicity study

**Figure 5:**
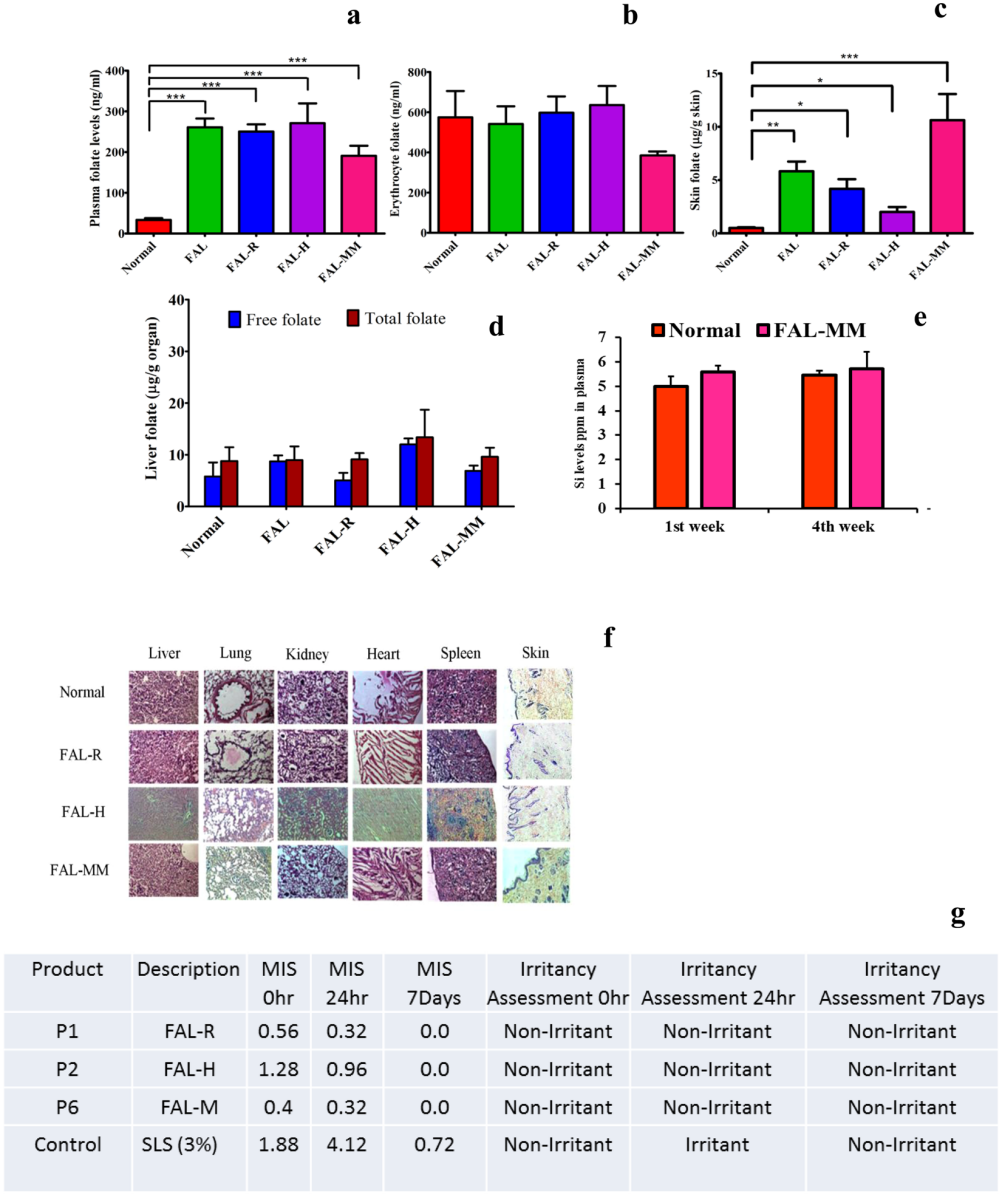
Sub-acute repeat dose and skin safety in rats (5 mg/kg FA dose/daily for 4 weeks) and Dermatological safety evaluation in 25 humans (Single application of RDA dose 400 µg/person). (**a**) Plasma profile in normal rats with significant folate levels due to Nutricosmetics. (**b**) Erythrocyte folate levels. (**c**) Skin folate levels showing reservoir of FA after 4 weeks. (**d**) Liver folate levels shows no toxicity in liver. (**e**) No significant Silicon levels(ppm) in plasma after 28 days daily FAL-MM application proves safety of FAL-MM. (**f**) Histopathology of H&E stained organs of rats sacrificed after 4 weeks observed under 10X. Normal group given regular diet throughout study whereas other groups were given folate deficient diet though out the study duration (**a**–**f**) N = 6 Mean ± SD ***P < 0.001, **P < 0.05 and *P < 0.01, Student’s t-test. (**g**) Skin compatibility dermatological evaluated of Nutricosmetics in humans shows no irritation potential for topical application in human volunteers.

To assess the safety of transdermal FAL, we conducted the sub-acute repeat dose toxicity study at a high dose level (5 mg/kg) as per OECD-420 and toxico-kinetics guidelines^18^. As depicted in Fig. 5a, biochemical parameters like plasma folate levels were found to be significantly higher than that of normal rats, but no adverse effects or behaviour in rats was seen. The erythrocytes and liver (free and total) folate levels confirmed the same (Fig. 5b,d). Skin depot with FAL-MM was significantly high compared to normal skin (P < 0.001) as observed in Fig. 5c. The haematological parameters were also found to be statistically similar with that of normal rats. Histopathological examinations of major organs revealed no abnormalities and no signs of any toxicity (Fig. 5f). Hence, it can be concluded that the cosmetics were safe as carriers for FAL as a transdermal application. Penetration of cosmetic through daily application was also monitored through systemic levels of silicate (Fig. 5e). The difference in silicon values in serum was found to be non-significant during depletion and repletion period. This confirms the inertness and safety of cosmetics for FAL-MM.

### Dermatological safety study on healthy human volunteers

Clinical assessment of standard single application patch test study BIS (The Bureau of Indian Standards) for nutricosmetics conducted on 25 healthy humans and was conducted by AIMS Research Pvt. Ltd. after ethical approval. The study duration was for 10 days and the skin was evaluated by a dermatologist as per Draize scale for erythema and edema in addition to wrinkles, scales, and volunteer’s response to the feeling of cosmetics (Fig. 5g). Single patch test showed no skin irritation potential of the folate loaded cosmetics.

## Discussion

In the quest to find an alternate to oral folic acid delivery, FA-loaded liposomes incorporated in cosmetics aimed at transdermal delivery and systemic absorption of folic acid for chronic treatment. The study demonstrated the proof of concept of feasibility of transdermal delivery of folic acid using liposomes incorporated in cosmetics. The unique composition of soya phosphatidylcholine and oleic acid incorporated in cosmetic bases showed enhanced transdermal penetration and high room temperature stability. This was a significant improvement over existing technology of liposomes which require refrigeration or lyophilisation for stability. The technology also overcame the disadvantages associated with the addition of synthetic surfactants and ethanol as permeation enhancers due to their potential for irritation and toxicity particularly on long term use.

Water soluble folic acid alone with log P value of −2.5 has negligible permeation across stratum corneum (1.98 ± 0.42 µg/cm^2^). No studies have been reported in the published literature for the transdermal delivery of folic acid using liposomes. Zeolite Y/alginate^19^ and calcium alginate^20^ hydrogels carrying folic acid as model active are one of the few studies that have attempted to deliver folic acid transdermally. The study was limited by poor penetration which required electroporation for enhancement. This process involves the additional cost of machinery^21^ and technical expertise which limit the widespread and long term use of the technology. Folate nutrition is required for long term therapeutic effect. While transdermal delivery of certain drugs has been studied using microemulsions, nanostructured lipid carriers or elastic liposomes, these have utilised ethanol and synthetic surfactants with or without electroporation for permeation. All the liposomes required lyophilisation or refrigeration to maintain stability. The present approach of nanosized liposomes containing phosphatidylcholine and fatty acids within cosmetic matrices overcomes the stability issue while enhancing the transdermal penetration. Manipulating and tweaking the liposome lipid content has strongly influenced its penetration across the skin to give desired targeted delivery. Fluidized liposomes demonstrate a better penetration and skin interaction compared to conventional and rigid liposomes as reported in literature for other drugs^22,23^. Oleic acid, an unsaturated fatty acid, has a low transition temperature compared to stearic acid, and the kink in the cis-alkenyl chain of oleic acid enhances its fluidizing effects and penetration through a deeper layer of skin Incorporation of oleic acid in a nano-sized liposome free of cholesterol enhanced the fluidising effects while reducing the rigidity associated with cholesterol added liposomes.

Our studies demonstrated that 9:1 w/w ratio of SPC-OA was effective in fluidizing the stratum corneum lipids with increased skin hydration. FAL of 120 nm size incorporated in various cosmetics lowered the permeation barrier with increased systemic folate levels. The systemic folate levels were sufficient to reverse *in-vivo* depleted folate reservoirs post application in folate-deprived rats. Albeit, the barrier was modulated for FA, the permeation of the cosmetic base ingredients was negligible. Dermatology sensitivity testing on humans further proved the safety of the Nutricosmetics. We report here for the first time, successful transdermal delivery of FA through cosmetics.

The technology acts as a platform for the non-invasive delivery of micronutrients for fortification. The advantage of using cosmetic bases not only enhances the room temperature stability of the liposomes but also does not require any behavior modifications in the users over long term and hence expected to increase compliance amongst girls and women. The presence of the cosmetic matrix stabilizes the liposomes for room temperature storage making them accessible in the developing world and in rural areas.

This technology is an innovative, patient-friendly and safe alternate strategy for women and adolescent girls of child bearing age, to increase compliance and build folate levels. To ensure affordability and a wider outreach of these cosmetics, natural and inexpensive GRAS-approved lipids like SPC and OA were utilized. FA is generally given as oral tablets containing 5 mg FA daily. An equivalent dose of FA is incorporated in all the cosmetics developed. 0.5 g Henna can cover both the palms of the hand, 1 g Face pack can be used daily as a cleansing agent without any side effects and 5 mL rose oil lotion can be applied on body or face skin. These everyday use volumes would be sufficient to deliver the required daily doses of folic acid. The costs of the folate-loaded cosmetics equivalent to 5 mg folate administration are expected to be 6 cents/application/day. The technology offers promise for transdermal fortification of folate and can be extended to other micronutrients also. Future clinical trials will give a clear idea of the long-term effects of transdermal folate fortification.

In conclusion, we have demonstrated transdermal folate loaded nutricosmetic as an effective folic acid fortification strategy. From a technological viewpoint, it allows the stabilisation of liposomes at room temperature and enhances transdermal penetration without requiring synthetic surfactants, ethanol or electroporation. From the application viewpoint, the dual technology of nutrient delivery-cum-beautification is also feasible in terms of its affordability and can be expanded for other nutrient delivery for wider applicability. The ease of use and incorporation into products of regular use is expected to improve the compliance aiding existing fortification strategies to augment folate levels in women globally.

## Methods

### Preparation of liposomes

Liposomes were prepared by thin film hydration scheme^24^ Different lipid to folic acid ratio liposomes was prepared (Fig. 1b). Blank liposomes of SPC, with oleic acid (SPC-OA), and stearic acid (SPC-SA) were prepared similarly without FA in the hydrating medium. For Rh-B loaded liposomes, the thin lipid film was rehydrated with Rh-B dye dissolved in phosphate buffer saline (0.2 mg/mL, pH 7.4). For fluidization and permeation studies, the liposome lipid dispersion after preparation was maintained at 20 mg/mL.

### Preparation of Nutricosmetics

FAL was incorporated in different cosmetics by vortexing to form a smooth spreadable paste and compared with a viscosity of commercially available products. FAL-H (FAL: henna powder::2 mL:1 g), FAL-MM (FAL: fuller’s earth::2 mL:1 g), FAL-R (FAL: rose oil::2 mL::0.1 mL) were prepared. Concentrated FAL dispersion was directly used as lotion using rose oil (FAL-R) as a fragrant. Cosmetics without FAL served as negative control. The viscosity of liposomal formulations was measured using parallel piped assembly in Anton Paar Rheoplus (Brookfield Engineering Lab, Incorporation; Middleboro).

### Fluidization of stratum corneum by chemical enhancers

Monolayer studies were performed on software controlled Langmuir Blodgett film balance with the model specification of KSV Mini trough, KSV Instruments, purchased from Finland. This apparatus consists of a Teflon trough and two Delrin barriers that are ideal for spreading of an insoluble lipid monolayer on the surface of subphase, filled in the trough, MilliQ in our experiment. The teflon trough is temperature controlled to maintain subphase temperature near to *in vivo* parameters, by circulating water around the teflon trough in form of a water jacket, at a temperature of 37 °C. An environmental chamber encloses the Langmuir apparatus to facilitate minimal disturbance to the formed monolayer. The Delrin barriers compress and expand the monolayer formed on the subphase. The change in pressure due to compression and expansion of monolayer was detected by wilhelmy plate balance, connected to pressure detection unit called force transducer. The change in pressure is automatically stored in software and was used for further analysis of other parameters like lift off limit^25^.

25 μl of skin tissue lipid extract in chloroform and its combination with SPC and SPC-Oleic acid/Stearic acid was thinly spread over the subphase surface with a Hamilton syringe. A waiting time of thirty minutes was set for organic solvent to evaporate from subphase. A compression speed of 120 mm/min was set for compression of lipid monolayer on subphase using Delrin barriers. The change in surface pressure with time was plotted, called as isotherm. This continuous compression of the monolayer was conducted in triplicate. The maximum relative area change observed in isotherm due to compression of lipid monolayer is 70%.

### Interaction of liposomes with stratum corneum lipids

Fourier Transform Infrared Analysis (FTIR)^26^ Bruker, Germany 3000 Hyperion Microscope with Vertex 80 FTIR System to observe the effect of liposomes on skin fluidization for 24 hr at room temperature. Skin pieces were then rinsed with water to remove excess of formulation, blotted dry on filter paper, lyophilized and taken for ATR-FTIR spectrometry. The ZnSe crystal directly touched the epidermis and 65 spectra/sec at 16 cm^−1^ were conducted. Opus® 5.5 software was used for spectral analysis.

### *Ex-vivo* skin permeation

Permeation experiments were conducted employing Franz diffusion cells under occluded conditions to prevent skin from drying. Defatted rat skin was clamped between the two compartments– donor and receptor (filled with 6 mL and saline) such that the stratum corneum faces towards the donor compartment thermostated at 37 ± 1 °C. Donor compartment was filled with 100 µl FA solution/FAL-R/FAL-H/FAL-MM (equivalent to 100 µg of FA) or Rh-B dye loaded liposomes and kept under occluded conditions. Aliquots of the receptor fluid (1 mL) were removed through the sampling port in the receptor zone at set time intervals of 2, 4, 6, 8, 12 and 24 h and maintained at −20 °C until further analysis and replenished with saline^27^. Aliquots and skin depot were analyzed using UV-VIS spectroscopy at *λ*_max_ 256 nm.

### Permeation mechanism

Rh-B-loaded liposomes were applied to rat skin for permeation as described in above. Transverse sections of the treated area of skin were sliced using microtome (~10 µm thickness) to generate XZ-planar optical cross-sections and scanned under a confocal laser scanning microscope (Olympus IX 81 with FV 500) for fluorescence signals with an argon laser beam with excitation at 488 nm and emission at 560 nm for Rhodamine-B^28^. The depth of liposome penetration was calculated from the confocal images captured in the XY-plane of the sections (perpendicular to the plane of skin surface). The intensity of the permeated depth was detected using Fluoview software. Blank skin served for correcting auto-fluorescence.

### *In-vivo* performance of liposomes

#### Selection of Animals

The protocols for animal studies were defended and approved by the Animal Ethics committee of National Toxicology Center, Pune for skin irritation and efficacy studies (Registration No. 166), and Oriental college of Pharmacy, Sanpada, Navi Mumbai for Pharmacokinetic (OCP/CPCSEA/2012-13/IIT/01) and toxicokinetics (OCP/CPCSEA/2012-13/IIT/04). The Committee for Purpose of Control and Supervision of Experiments on Animals (CPCSEA) is a statutory Committee, which is established under Chapter 4, Section 15(1) of the Prevention of Cruelty to Animals Act 1960. CPCSEA guidelines were adhered during the entire maintenance and experimental period of animal studies. All the animal experiments were performed in accordance with relevant guidelines and regulations. Female Sprague Dawley rats (8–10 weeks/125–150 g) were supplied by Glenmark Pharmaceuticals, Navi-Mumbai respectively for pharmacokinetic and toxicokinetic studies. The animals were acclimatized under standard conditions in 12 h light/12 h dark cycle at 25 ± 2 °C and fed with recommended diet and water. A folate-deficient diet containing 1% w/w succinylsulfathiazole was procured from VRK Nutritional Solutions, Sangli.

#### Skin irritation study

The presence of any skin irritation was assessed after application of FA/FAL /FAL-R/FAL-H/FAL-MM on rat skin for 28 days. Rats were distributed into six groups each of six animals. Group served as animals treated with FA/FAL-R/FAL-H/FAL-MM. Formulations (1 mg/kg of FA, ~0.1 mL) were gently applied onto skin surface area of 3 × 3 cm^2^. After a daily application for 4 weeks, the formulations were wiped off, evaluated for any visible erythema and edema compared to Draize scale at 1 h, 24 h, 48 h and 72 h, 1st week, 2^nd^ week, 3^rd^ week and 4^th^ week post treatment. Skin irritation was scored as 0 if there was no reaction; 1 in cases of diffuse erythema and edema; 2 in cases of weak but noticeable erythema on or around the area of treatment; 3 in cases of moderate erythema; and 4 in cases of severe erythema with edema^29^.

#### Pharmacokinetic study

Experimental animals were divided into the 4 groups each containing four animals with their dorsal side shaved 18 h prior to the study and fasted over this period. Group A was kept as control, B, C and D received a single dose of transdermal FAL-R, FAL-H, and FAL-MM respectively. Folic acid dose equivalent to 5 mg/kg body weight was given. Blood samples (0.4 mL) were drawn at different time intervals of 0, 2, 4 and 8 h post-application. Plasma was separated by centrifugation (Sigma3K30) at 5000 rpm for 20 min, harvested into another tube containing 0.5% freshly prepared an L-ascorbic acid solution to prevent folate oxidation. The samples were stored at −20 °C until analysis for plasma folate by the chemiluminescence method (Centaur Advia, Siemens, India).

#### *In-vivo* efficacy study in Sprague Dawley rats

The animals were fed with folate deficient diet. After 6 weeks depletion period, rats were daily administered with different FA formulations for 4 weeks. The different groups (*n* = 6 animals per group) were group 1 as normal control group fed with standard diet and with no treatment, group 2: Anemic control group deprived of folate throughout the study with no treatment, group 3: Oral administration of FA solution (500 µg/kg FA), group 4–6: Transdermal application of FAL-R/FAL-H/FAL-MM (1 mg/kg FA), respectively. At end of four weeks repletion period, various hematological and plasma biochemical parameters were evaluated.

#### Repeat-dose toxicity in rats

A modified subacute 28-days repeat-dose dermal toxicity study was carried out to incorporate the toxico-kinetics and folate distribution in body reserves. The major storage reservoirs of folate are liver and erythrocytes. Regular folate systemic exposure was studied in folate deficient Sprague Dawley rats at lowest fixed dose level (5 mg/kg) as per OECD-420 and ICH Topic S3A Toxico-kinetics guidelines. The rats had *ad libitum* access to their respective diets and water during the 10-weeks test period. During the test period, body weight and plasma folate were monitored weekly. After 6 weeks of feeding folate depleted diet, rats were daily administered with different FA formulations for 4 weeks. The different groups were: group 1-normal control group fed with standard diet and with no treatment, group 2–5: transdermal application of FAL/FAL-R/FAL-H/FAL-MM (5 mg/kg FA), respectively. At the end of the stipulated treatment period of four weeks repletion, rats were anesthetized and bled by cardiac puncture into Vacutainers containing EDTA for hematology studies and erythrocyte folate levels. Hematology and erythrocyte folate levels were determined within 24 h of blood withdrawal. Livers were isolated, flushed and homogenized in 100 mM phosphate buffer (pH 4.8) containing 1% ascorbic acid^30^. Two sets of ten-time diluted homogenates were prepared. One set of homogenates was autoclaved at 120 °C for 5 minutes (to determine free folate in the liver) and the other set was incubated at 37 °C for 24 h followed by incubation (to determine total folate in the liver). Skin samples were also excised at the site of application. Skin sample of dimensions 1 cm × 1 cm were left overnight for extraction in 100 mM phosphate buffer (pH 4.8) containing 1% ascorbic acid. All extracts were stored at −20 °C until analysis for folate by a chemiluminescence assay (Centaur Advia, Siemens, India). Assessment of toxicity was based on body weight, clinical pathology, organ weight and histopathological examinations. Various vital organs (liver, lung, spleen, kidney, heart, and skin) of the above-euthanized rats were isolated and preserved in 10% neutral buffered formalin. Microtomed tissue sections were stained with hematoxylin and eosin. They were observed under 10X magnification of light microscope, photomicrographed and compared to the control group.

#### Dermatological safety study on healthy human volunteers

Single application, single centered, open label, closed patch test to evaluate the safety of cosmetic formulations on healthy human subjects representing varied skin types was conducted by AIM Research Services, Pvt. Ltd. Mumbai, Report Number: AIM-CRD-031-15. The study was registered with Clinical Trial Registry of India (CTRI) CTRI: REF/2015/12/010244 in accordance with relevant guidelines and regulations. The Bureau of Indian Standards (BIS) is the national Standards Body of India working under the aegis of Ministry of Consumer Affairs, Food & Public Distribution, Government of India. The Bureau of Indian Standards (BIS) 4011: 1997 methods of test of safety evaluation of cosmetics on humans was followed and approved by CTRI. Informed consent was obtained from all the participants prior to the study.

The study was conducted on 25 healthy humans (12 females and 13 males) after satisfying the inclusion and exclusion criteria with informed consent ranging in the age group 18–50 years over a period of 10 days. 40 µl/0.040 g of the test sample containing 400 µg of folate was dispensed on Whatman filter paper, placed within aluminum chambers, prefixed on fix pull tape and applied on the back between scapula and waist under occlusion for 24 h. A dermatologist examined the skin test Day 2 (Application of patch), Day 3 (Removal of patch and assessment for 30 min) Day 4 (reaction after 24 h) and Day 10 (reaction after 7 days). Sodium Lauryl Sulphate (SLS) at 3% Concentration was used as positive control. Draize scale (0- No reaction, 1- very slight, 2- slight, 3- moderate, 4- severe erythema/edema) was used to evaluate irritation response such as erythema (redness), edema (swelling) and vesiculation. Average Mean Score for Irritation (AMS) = {Total score (Erythema + Edema) for each sample}/Total no. of Subjects. Interpretation of Mean scores 2 (Non-irritant), 4 (Mild irritant) and 8 (Irritant). AMS is standard procedure to calculate irritation potential of the test substance on skin.

### Statistical analysis

All data are presented as a mean ± standard deviation for minimum triplicate measurements unless where specified. Statistical significance was determined by unpaired two-tailed student t-test with the confidence interval of 95% to determine significance*. P* < *0.05* was considered statistically significant.

## Electronic supplementary material

Supplementary data

## Data Availability

Data is available on request from the corresponding author.
