# Supplementary material for: Stable Liposome in Cosmetic Platforms for Transdermal Folic acid delivery for fortification and treatment of micronutrient deficiencies
Source: Sci Rep. 2018 Oct 31;8:16122. doi: 10.1038/s41598-018-34205-0 (PMC6208427; doi:10.1038/s41598-018-34205-0)
Supplement: Supplementary file 1 — Supplementary data [file 41598_2018_34205_MOESM1_ESM.docx]

**Stable Liposome in Cosmetic Platforms for Transdermal Folic acid delivery for fortification and treatment of micronutrient deficiencies**

Mudra Saurabh Kapoor^1^, Anisha D’Souza^1^, Noorjahan Aibani^1^, Swathi Sivasankaran Nair^1^, Puja Sandbhor^1^, Durga kumari^1^ and Rinti Banerjee^1^

^1^Department of Biosciences & Bioengineering, Indian Institute of Technology Bombay, Powai, Mumbai 400076, India

Correspondence to: rinti@iitb.ac.in

**Supplementary data**

Table 1: Encapsulation efficiency, particle size and surface charge of SPC-OA particles with different lipid to folate ratio. N=3, Mean ± SD Lipid: FA in the ratio 2:1 shows 120+4.1 nm size with stable surface charge and was selected for delivering folate through cosmetics

| Lipid: Drug  Concentration  (w/w) | Encapsulation Efficiency (%) | Particle size  (nm) | Zeta potential  (mV) | PDI |
| --- | --- | --- | --- | --- |
| 2:1 | 10 ± 1.60 | 120 ± 4.1 | -27 ± 3.1 | 0.33 |
| 4:1 | 70 ± 3.33 | 198 ± 0.2 | -14 ±2.5 | 0.22 |
| 6:1 | 40 ± 0.48 | 260 ± 0.4 | -46 ±6.5 | 0.28 |
| 8:1 | 6 ± 0.25 | 280 ± 0.4 | -36 ± 2.6 | 0.14 |

Figure 1: Elemental composition of Fuller’s earth analyzed by EDAX

Table 2: Nutricosmetic composition and viscosity data. N=3. Mean+SD.

| **Nutricosmetic** | **Cosmetic:FAL** | **Viscosity Pa.S**  **Day 1** | **Viscosity Pa.S**  **After 6 months** |
| --- | --- | --- | --- |
| FAL-R | 0.6:1(v/v) | 21 ± 3.5 | 26±2.4 |
| FAL-H | 2.5:1(w/v) | 396 ± 15.8 | 414±21 |
| FAL-MM | 1.5:1(w/v) | 28± 8.3 | 32±6.5 |


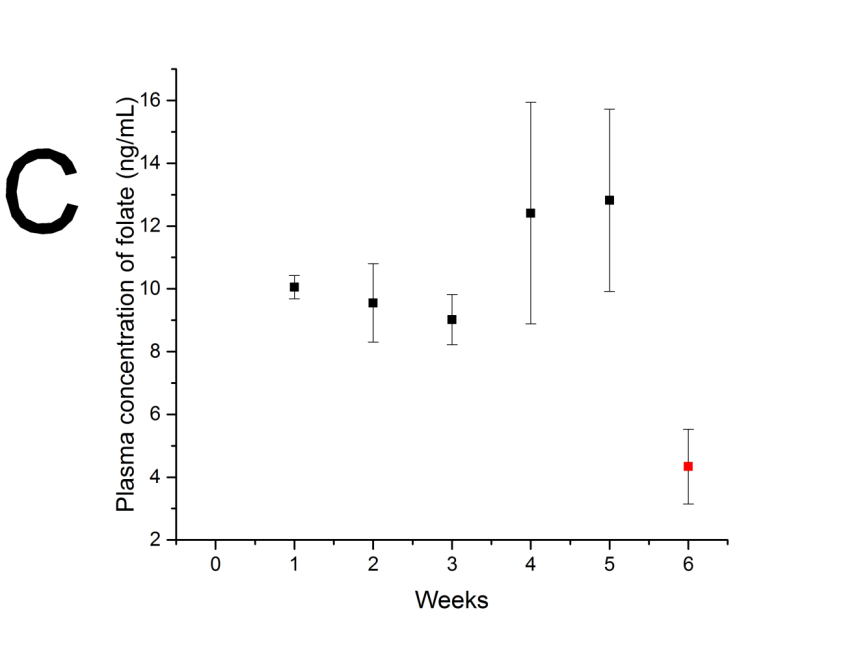


Figure 2: Folic acid deficiency model induced in Sprague dawley rats. Red color reading signifies the anemic condition of rats at 4ng/mL. Mean + SD, N=30.
